# Seasonal dynamics and nutrient controls of biogenic silica in Baltic Sea surface microplankton and picoplankton communities

**DOI:** 10.1128/aem.00676-25

**Published:** 2025-04-28

**Authors:** Yelena Churakova, Anabella Aguilera, Evangelia Charalampous, Daniel J. Conley, Daniel Lundin, Jarone Pinhassi, Hanna Farnelid

**Affiliations:** 1 Centre for Ecology and Evolution in Microbial Model Systems (EEMiS), Linnaeus University 4180, Kalmar, Sweden; 2 Department of Geology, Lund University 225265, Lund, Sweden; Colorado School of Mines, Golden, Colorado, USA

**Keywords:** marine silica cycle, time series, microcosm, biosilicification

## Abstract

In recent years, new contributors to the marine silica cycle have emerged, including pico-sized phytoplankton (<2–3 µm in size) such as *Synechococcus* and picoeukaryotes. Their contribution and relevance to silica cycling are still under investigation. Field studies reporting the biogenic silica (bSi) standing stock in the pico-sized fraction are limited to silica-poor oligotrophic environments, and the mechanism of bSi accumulation in picoplankton remains unknown. We investigated the variability of bSi standing stocks in two size fractions (picoplankton, 0.22–3 µm and microplankton, >3 µm) in the dissolved silica-replete Baltic Sea via biweekly time series samplings spanning 2 years. Time series data showed that the large changes in bSi standing stock in the Baltic Proper were primarily related to microplankton biomass and community composition. Meanwhile, picoplankton were, at times, surprisingly high contributors to total bSi year-round (up to 21.6%). Simultaneously, we performed microcosm incubation experiments with natural phytoplankton communities in each season to examine how nutrient additions affected bSi concentrations. In these experiments, increases in microplankton bSi were directly correlated to increases in diatom biomass, highlighting their influential role in the Baltic Sea silica cycle. Meanwhile, phosphorus additions triggered an increase in picoplankton bSi accumulation in all experiments. This uncovers a potential control of bSi accumulation in picoplankton, which can help identify the cellular mechanisms behind this process and uncover their role in silica cycling. The results link phytoplankton community composition and silica cycling, which is important for understanding the consequences of organism shifts due to climate change.

**IMPORTANCE:**

The marine carbon and silica cycles are tightly intertwined and largely controlled by diatoms. Nevertheless, recent studies, mostly in oligotrophic waters, have proposed new contributors to the marine silica cycle: picoplankton. Here, we report the first study of seasonal dynamics of biogenic silica (bSi) standing stock in microplankton and picoplankton in the silica-replete Baltic Sea. Microplankton bSi dynamics were correlated with changes in composition and biomass. Picoplankton were consistent contributors to bSi, and for the first time in diverse natural communities, we found a direct correlation between phosphorus and bSi accumulation. The results are important for understanding how climate change-predicted phytoplankton composition shifts will affect carbon and silica cycling and provide a direction for future research on nutrient controls of silica accumulation in picoplankton.

## INTRODUCTION

Marine silica cycling is characterized by constant fluctuations between the lithosphere and hydrosphere, which are governed by a variety of abiotic and biotic sources and sinks. Diatoms are a major biological component of this system, producing biogenic silica (bSi) frustules from silicic acid (dissolved silica, dSi) and recycling it in the euphotic zone. These frustules also enable their sinking to other oceanic zones and, eventually, ocean sediments, which couples the marine carbon and silica cycles (1). Biosilicifying eukaryotic groups such as sponges and Rhizaria are also known for their contributions to the marine silica cycle, though they are estimated to be less important than diatoms (1–3). Besides these relatively well-studied groups of silicifying organisms, research over the past decade has also begun to shed light on a previously understudied biotic component of the marine silica cycle: nontraditional biosilicifiers. Nontraditional biosilicifiers accumulate bSi despite lacking siliceous cell structures and can be composed of picoplankton (<2–3 µm in diameter) such as the picocyanobacterium *Synechococcus* (4, 5), picoeukaryotes (6), as well as selected calcifying coccolithophore species (7, 8) and also larger plankton (9). Some of these plankton groups have genes involved in the uptake and/or assimilation of dSi (e.g., silicon ion transporters), while others do not (9). Due to the lack of knowledge about how non-silicified organisms utilize and accumulate dSi, the contribution of nontraditional biosilicifiers to marine silica cycling is unclear.

The biotic component of the marine silica cycle is estimated to produce 255 (±52) Tmol silicon year ^−1^ (1). Prior to the past decade, knowledge about biotic contribution was largely based on studies of bSi production and stocks performed without the use of size fractionation. Thus, bSi associated with picoplankton was indistinguishable from that of larger phytoplankton, even though filters with small pore sizes were used to measure bSi (typically 0.6 µm or greater [10]). Since the discovery of bSi accumulation in *Synechococcus* isolates from the Sargasso Sea (4), several studies have measured bSi standing stocks (quotas) and Si production rates in the pico-sized fraction. In the regions studied, including the North Atlantic Ocean, Sargasso Sea, South Pacific Ocean, North Pacific Ocean, Eastern Indian Ocean, and Western Pacific Ocean, estimates of *Synechococcus* contribution to total bSi standing stock range from approximately 1% to 66% (11–16). Thus far, these studies have been conducted in dSi-limited oligotrophic ocean gyres (with the exception of a single station located in a nutrient-rich upwelling system in reference 11) with the rationale that diatom biomass is low and picoplankton biomass is high in these regions, making signals from picoplankton relatively more important and easier to identify. The majority of these studies have been performed on research cruises to investigate the spatial distribution of bSi production (measured indirectly as silicic acid uptake) and stock, and few examine long-term (e.g., seasonal and interannual) silica cycling dynamics in marine environments (17). Our understanding of silica cycling is particularly limited regarding picoplankton, and there is no definitive link with specific environmental or biological factors, suggesting that studies involving diverse environments and expanded timescales are needed.

The Baltic Sea is a large, semi-enclosed, brackish water body divided into distinct regions characterized by north to south gradients in salinity, nitrogen, and phosphorus. The south, stretching from the Kattegatt to the Baltic Proper, is characterized by nitrogen limitation in the summer and medium to high salinities (7 to >20 PSU). The north, consisting of the Bothnian Bay and Bothnian Sea, is characterized by phosphorus limitation in the summer and low salinity (3–6 PSU [18]). Dissolved Si concentrations in surface waters in the southern Baltic average 10–20 µmol L^−1^ (19), which are significantly higher than dSi concentrations typically measured in oligotrophic ocean surface waters. In most regions, dSi concentrations are not deemed as limiting for diatom growth (19, 20) and are stable or increasing in both coastal and open waters (21). Though there have been studies of bSi in sediments (22–25), there are no published studies of bSi standing stock dynamics in the surface waters of the Baltic Sea, highlighting a major gap in our understanding of silica cycling in this environment.

The composition of Baltic Sea surface water plankton communities varies throughout the year, adjusting to shifts in temperature, light, nutrients, and other seasonal factors (26, 27). We know that picophytoplankton (predominantly picocyanobacteria, including a mix of *Cyanobium* spp. and *Synechococcus* spp. strains and photosynthetic picoeukaryotes [PPE]) make up a substantial portion (15%–85% of chlorophyll *a* as a proxy for biomass) of the Baltic Sea phytoplankton community (28–31). In the Baltic Proper, picophytoplankton are dominant features of both coastal and offshore phytoplankton communities throughout the year, only lowering in abundance when the temperature falls <5°C (32). Furthermore, picophytoplankton biomass is forecasted to increase due to climate change, while, in contrast, warmer winters are predicted to dampen the intensity of diatom blooms (33). In the Baltic Sea, the pronounced shift to dinoflagellate-dominated spring blooms, replacing diatom-dominated ones, has been noted (34–36). As a result of this shift in plankton community dynamics, nutrient cycles can be expected to be altered (e.g., through differences in the utilization of nutrients like dSi) with cascading effects, as detailed extensively in reference 36. Several photosynthetic picoeukaryote and *Synechococcus* isolates from the Baltic Proper have demonstrated bSi accumulation in laboratory experiments (6, 37). This suggests that picophytoplankton are players in the Baltic Sea silica cycle, though their contribution is currently unknown.

To fill this knowledge gap, we investigated bSi standing stock (herein referred to as bSi) dynamics of picoplankton and microplankton fractions (0.22–3 µm and >3 µm) at a sampling station in the Baltic Proper by integrating data from time series and microcosm experiments. The focus of our study was the picoplankton fraction. The time series data, collected over the course of 2 years, depicted the seasonal shifts in bSi and the contributions of the two size fractions to total bSi. The microcosm experiments, conducted in four different seasons, in which we enriched nutrient availability, revealed differences in environmental and biological factors influencing bSi accumulation in the two size fractions.

## MATERIALS AND METHODS

### Field sampling

Sampling was carried out at the Linnaeus Microbial Observatory (LMO), located 11 km offshore off the east coast of Öland, in the Baltic Proper (N 56° 55.8540′, E 17° 3.6420′; Fig. S1). Temperature and salinity were measured using a CTD probe (AAQ 1186-H, Alec Electronics) from June 2021 to November 2022 and then with a CastAway-CTD (SonTek, Xylem) from December 2022 to June 2023. Water samples were collected from 2 m depth for two purposes: bSi dynamics in an ongoing twice-monthly LMO time series (previous publications include references 26, 27), and a total of four microcosm experiments to test factors influencing bSi accumulation in different seasons. Over the course of a year (2022–2023), microcosm experiments (herein referred to as SPR, SUM, AUT, and WIN) were performed (Table 1). Upon arrival at the laboratory, the water was filtered through a 200 µm mesh into a 210 L opaque polypropylene mixing container to minimize the effect of larger grazers. Experimental setup and sample processing followed shortly thereafter.

**TABLE 1:** Microcosm experiment information including the temperature (°C) and salinity (PSU) of seawater measured at the time of sampling, initial (t0), and final time points after 65 hours (t65)^*a*^

Microcosm	Temperature (°C)	Salinity (PSU)	Initial time point (t0) date	Final time point (t65) date	Light:dark hours
SPR	4.7	7.9	11 April 2022	14 April 2022	13:11
SUM	18.4	7.5	18 July 2022	21 July 2022	18:6
AUT	8	7.7	18 October 2022	21 October 2022	12:12
WIN	3.4	7	7 February 2023	10 February 2023	9:15

### Microcosm experimental setup

In total, there were six treatments (control [no addition], 1.6 µmol L^−1^ ammonia [NH_4_], 5 µmol L^−1^ nitrate [NO_3_], 1.5 µmol L^−1^ phosphate [PO_4_], 1.6 µmol L^−1^ NH_4_ + 1.5 µmol L^−1^ PO_4_, and 5 µmol L^−1^ NO_3_ + 1.5 µmol L^−1^ PO_4_) with three biological replicates. The added nutrients were approximately double the average winter nutrient concentrations at LMO, based on historical data (26). To set up the experiment, acid-washed 4.7 L Nalgene polycarbonate bottles were filled with 4.5 L of sample water, and nutrients were added according to each treatment. The bottles were incubated in a shaded tank with continuously circulating seawater, under 60 µmol photons m^−2^ s^−1^ irradiance for 65 hours from the initial (t0) to the final time point (t65). The shading percentage was determined by calculating the average difference between light intensity at the surface versus 2 m depth using historical LMO data. The light:dark hours were adjusted in accordance with the calculated light hours (in between sunrise and sunset) of the sampling date (Table 1). The bottles were manually mixed daily during the incubation period. Samples were collected at t0 and t65 from the whole water for nutrient analysis, microscopy, flow cytometry, heterotrophic bacterial production, and primary production. Meanwhile, chlorophyll *a*, bSi, and DNA were sampled in two size fractions (picoplankton, 0.22–3 µm and microplankton, >3 µm).

### Abiotic parameters

For the time series, samples for dissolved inorganic nutrients (nitrite + nitrate [NO_2_ + NO_3_], ammonium [NH_4_], phosphate [PO_4_], and dSi [SiO_2_]) were collected, frozen at −20°C, and later measured using a UV-1600PC Spectrophotometer (VWR) and DR 5000 (Hach Lange) following standard protocols (38). For the microcosm experiments, the samples were sent to ALS Global for analysis under the guidelines of the “Nutrient salts in clean water package.” Detection limits for the measured nutrients were NO_3_ (0.026 µmol L^−1^), NO_2_ (0.033 µmol L^−1^), NH_4_ (0.125 µmol L^−1^), total N (1.428 µmol L^−1^), PO_4_ (0.008 µmol L^−1^), and total P (0.097 µmol L^−1^). For dSi analysis in microcosms, a 10 mL sample was passed through a 33 mm diameter 0.22 µm pore-size Millex-GP filter (Millipore) and stored at −20°C before analysis following reference 39. We have previously tested the reliability of the freeze-thaw method to analyze dSi samples and found that we obtain the same values before and after freezing (6). The measurements were done with a UV-1600PC spectrophotometer (VWR). Biogenic silica standing stock was measured following the protocols outlined in references 5 and 40. Depending on the season, 300–800 mL of seawater was filtered through successive 47 mm diameter 3.0 µm and 0.22 µm pore-size Nuclepore Track-Etch filters (Whatman). For the time series, bSi was measured in duplicate, and for the microcosm experiments, there was one technical replicate for each of the three biological replicates. The filters were stored in 2 mL Eppendorf tubes at −20°C until analysis, which included a digestion step in 0.2 M NaOH at 95°C for 1 hour. Biogenic Si measurements were done at 810 nm using a UV-1600PC spectrophotometer (VWR) using 50 mm cuvettes. We note that, technically, when bSi is extracted via the NaOH method, some lithogenic silica (LSi) can also be dissolved, making the measurement a measure of particulate silica (41). The LMO sampling station is offshore and therefore likely less influenced by river and groundwater inputs, which are the main sources of LSi in surface estuarine waters (42, 43) (Fig. S1). However, to verify that the influence of LSi was minor, we measured LSi in five samples across the time series. The LSi concentrations measured (0.017–0.076 μmol L^−1^; Table S1) were lower than typically seen interfering with measurements from oligotrophic regions (44), and would not alter the general trends in bSi that we observed.

### Biotic parameters

Extracting and measuring chlorophyll *a* was done following the protocol by Jespersen and Christoffersen (45). For the time series, 500 mL of seawater was filtered through glass fiber (A/E) filters under low vacuum in duplicate. During the microcosm experiments, 400–800 mL (t0: five replicates and t65: one replicate of each biological triplicate) was filtered through successive 47 mm diameter 3.0 µm Nuclepore Track-Etch (Whatman) and precombusted (475°C, 2 hours) GF/C glass fiber (Advantec) filters. The filters were extracted overnight in 5 mL of 96% ethanol, and fluorescence was measured the following day with a fluorometer (Turner design Model #040). For determining picophytoplankton and bacterial abundances, samples were fixed with glutaraldehyde solution Grade 1 (1% final concentration; Sigma-Aldrich) in Cryovial tubes (Sigma-Aldrich) and frozen at −80°C. A CyFlow Cube 8 (Partech) flow cytometer with a blue (488 nm) and a red (638 nm) laser set to a flow rate of 10 µL s^−1^ was used for analyzing LMO samples collected prior to July 2022 and for SPR and SUM samples. All other flow cytometry samples were analyzed with a CytoFLEX (Beckman Coulter) flow cytometer with a blue (488 nm) and a red (638 nm) laser. The samples were analyzed for two populations of picophytoplankton (photosynthetic picoeukaryotes and picocyanobacteria) using the gating strategy described in reference 46. Samples for determining bacterial abundance were stained with SYBR Green I nucleic acid stain (Invitrogen; 5 µM final concentration) and counted as advised by Gasol and Del Giorgio (47). Flow cytometry-related gating and visualization were done using FCSalyzer version 0.9.22-alpha or CytExpert version 2.5.0.77, depending on the machine used for the sample analysis. The mean of technical duplicates for each biological replicate was calculated.

### Primary and bacterial production

Measuring primary production in microcosm samples was done using the ^14^C incorporation method. Briefly, 10 mL samples were incubated with 6 µL (1 µCiµL^−1^ working solution) of ^14^C-bicarbonate (1 mCi mL^−1^; Perkin Elmer). Two technical replicates (one incubated in the light and one in the dark) per biological replicate (t0: four technical replicates of light incubation and two of dark; t65: one replicate of each biological triplicate) were incubated for 2 hours at *in situ* temperature conditions in the incubation tank and subsequently filtered onto 25 mm black polycarbonate 0.22 µm Nuclepore Track-Etch filters (Whatman). Analysis was done with a liquid scintillation counter (Tri-Carb 2100TR, Packard), measuring the amount of ^14^C incorporated by cells (48). Measuring bacterial production in microcosm samples was done following reference 49 by first diluting commercial ^3^H-leucine (specific radioactivity 153 mCi mL^−1^; Perkin Elmer) with cold leucine for a working 1 mM solution (50). Four technical replicates and two killed controls (trichloric acid; 5% final concentration; Sigma-Aldrich) per biological replicate (t0: one replicate and t65: one replicate of each biological triplicate) were incubated with the diluted ^3^H-leucine (40 nM final concentration) for 2 hours at *in situ* temperature. A liquid scintillation counter (Tri-Carb 2100TR, Packard) was used to measure the amount of radioactivity incorporated by cells. Values, including a cellular carbon-to-protein conversion factor of 0.86 kgC mol leu^−1^, assumed proportion of leucine in total protein of 0.073%, and isotopic dilution factor of 2, were used to calculate bacterial production (51). The mean of four technical replicates for each biological triplicate was calculated.

### Phyto- and bacterioplankton biomass and community composition

To identify and count phytoplankton (t0: one replicate; t65: one replicate of each biological triplicate), samples were fixed with Lugol’s solution (1% final concentration) and analyzed in sedimentation chambers with an inverted microscope (Nikon TMS) following the Utermöhl method. Cells were counted and identified to genus or species level, and the biomass was calculated based on the biovolume (52) and carbon content (53, 54). For DNA sampling, up to 1.4 L for every sample (t0: five replicates; t65: one replicate of each biological triplicate) was filtered through successive 47 mm 3.0 µm Nuclepore Track-Etch (Whatman) and 0.22 µm Sterivex cartridge filters (Millipore) using a peristaltic pump. The Sterivex filters for DNA analysis were frozen in TE buffer at −80°C until extraction. For DNA extraction, the FastDNA SPIN Kit for Soil (MP Biomedicals) was used according to the manufacturer’s protocol, with the addition of a 1-hour proteinase-K (0.02 µg µL^−1^, final concentration) incubation at 55°C. The V3V4 region of the 16S rRNA gene, using primer pair 341F-805R (55), was amplified with PCR using Thermo Fisher Scientific Phusion High-Fidelity PCR Master Mix manufacturer instructions. Sequencing was done at the National Genome Infrastructure, SciLifeLab Stockholm, using a MiSeq platform (Illumina), producing 2 × 300 bp paired-end reads.

### Bioinformatic and statistical analyses

The 16S rRNA gene amplicon data were processed using the Ampliseq pipeline (version 2.8.0 [56]) from the nf-core framework (57). Ampliseq is a Nextflow (58) pipeline that uses DADA2 (version 1.28.0 [59]) to denoise and assign taxonomy to amplicon sequences. It uses Cutadapt (version 3.4 [60]) to remove primer sequences and Barrnap (version 0.9, https://github.com/tseemann/barrnap) to validate small subunit rRNA gene sequences. Taxonomy assignment was done by combining the SILVA 132 (61), SBDI-GTDB (R07-RS207 [62]), and Phytoref (63) databases. To assign eukaryotic taxonomy, if the confidence scores of ASV sequences identified as chloroplasts by SILVA 132 were higher in the Phytoref database than in the SILVA 132 database, then Phytoref taxonomic assignment was used.

Statistical analyses were done, and figures were created with RStudio version 2023.06.0+421 (64). The *tidyverse* and *ggplot2* packages were broadly used for visualization. Kruskal-Wallis ANOVAs and *post hoc* Dunn tests were performed with base functions in R. Packages *Hmisc* and *corrplot* were used to calculate the significance and visualize results from a Spearman correlation analysis. Package *vegan* (version 2.6.4) was used to calculate dissimilarity indices for PCoAs. Package *ALDEx2* was used to do a CLR transformation of the 16S count data. PCoAs and PCAs were performed on relative abundance microscopy data and CLR-transformed 16S relative abundance data, respectively, in order to assess nutrient treatment effects on community composition.

## RESULTS

### Field observations

**Fig 1:**
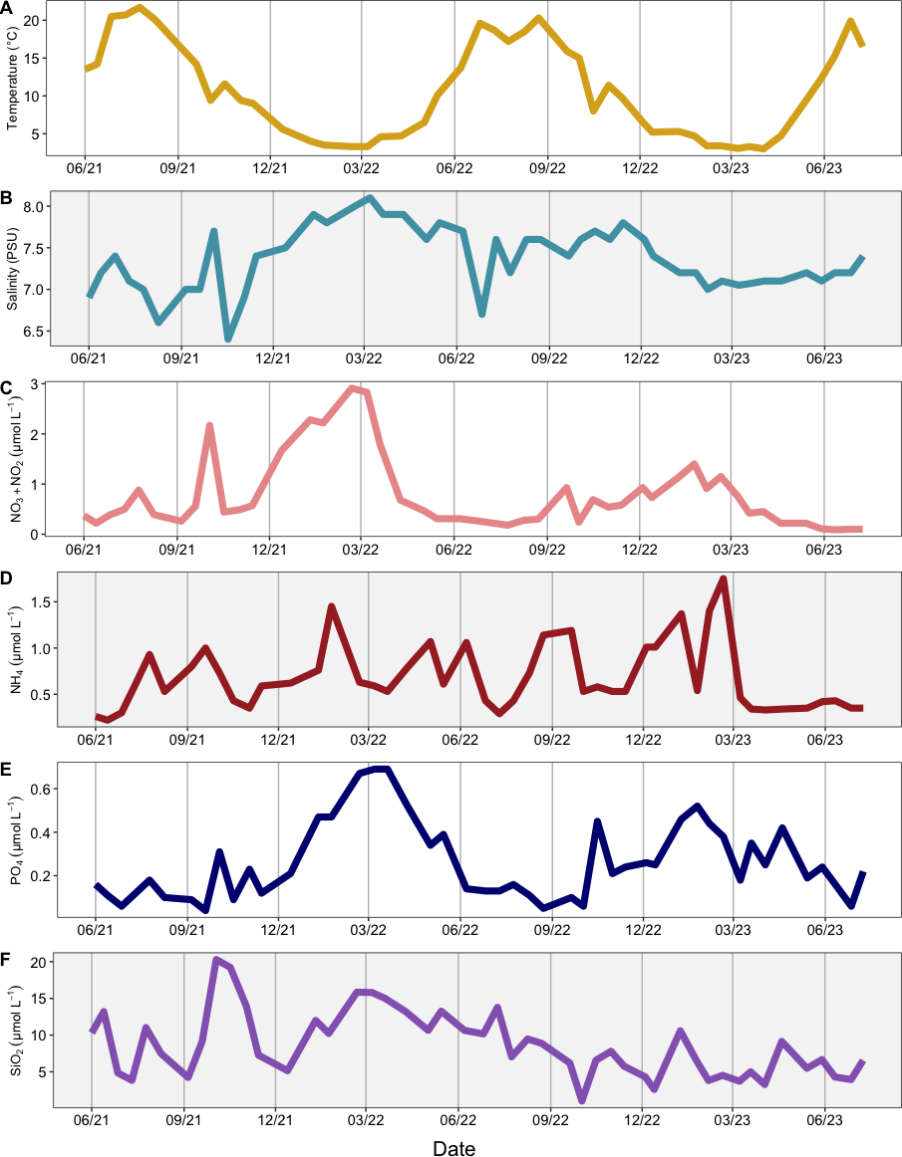
Environmental parameters measured at LMO from June 2021 to June 2023. These parameters include (**A**) temperature (°C), (**B**) salinity (PSU), (**C**) NO_3_ + NO_2_ (µmol L^−1^), (**D**) NH_4_ (µmol L^−1^), (**E**) PO_4_ (µmol L^−1^), and (**F**) SiO_2_ (dSi) (µmol L^−1^).

**Fig 2:**
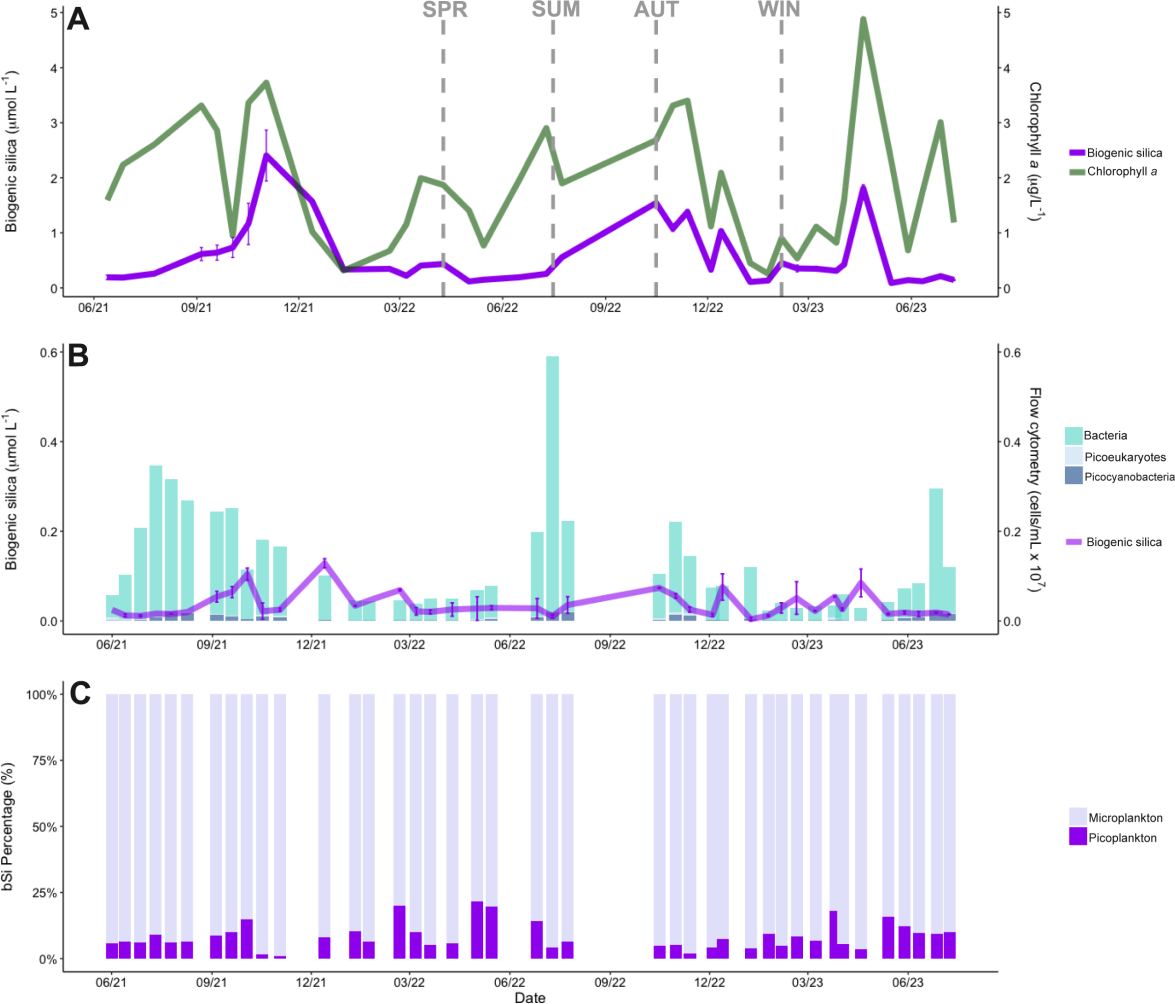
Variation of bSi, chlorophyll *a*, and picoplankton abundances in the Baltic Sea during the study period showing (**A**) total bSi standing stock (µmol L^−1^) plotted against total chlorophyll *a* (µg L^−1^) concentrations, and (**B**) bSi standing stock (µmol L^−1^) in picoplankton plotted against flow cytometry total cell counts (cells mL^−1^ × 10^7^) of heterotrophic bacteria, PPE, and picocyanobacteria populations, and (**C**) the proportions of the contribution of the microplankton (light purple) and picoplankton (dark purple) to total bSi. Dashed gray lines (**A**) indicate the sampling dates of the microcosm experiments. Error bars in the line graphs represent the standard deviations from the means based on two technical replicates.

Water from the LMO station was sampled approximately twice a month from June 2021 to June 2023, resulting in 41 available data sets. During this time, temperature ranged from 3.0℃ to 21.7℃ (Fig. 1A). Salinity varied from 6.4 to 8.1 PSU, with higher and more stable concentrations in autumn and winter (Fig. 1B). The highest NO_3_ concentrations (measured as NO_3_ + NO_2_) were in autumn and winter and were otherwise low in spring and summer (Fig. 1C). PO_4_ concentrations exhibited similar patterns to NO_3_ (Fig. 1E), while NH_4_ varied without a consistent pattern (Fig. 1D). Concentrations of SiO_2_ ranged from 1.0 to 20.3 µmol L^−1^ (Fig. 1F). Total chlorophyll *a*, used as a proxy for phytoplankton biomass, changed considerably over time (~0.3–4.9 µg L^−1^), with low concentrations in the winter and peaks indicating phytoplankton blooms of varying intensities in the spring, summer, and autumn (Fig. 2A). Total bSi (µmol L^−1^), the sum of bSi in the microplankton and picoplankton, also fluctuated considerably throughout the years (~0.09–1.81 µmol L^−1^; Fig. 2A). The highest total bSi values were measured in autumn, though a significant peak was also observed during spring 2023. The maximum value for picoplankton bSi was 0.12 µmol L^−1^, measured in winter 2022 (Fig. 2B). Picoplankton accounted for 1.1%–21.6% of the total bSi (Fig. 2C). In spring and summer 2022, as well as spring 2023, several samplings with higher contributions of picoplankton to bSi (>20%; Fig. 2C) were observed.

**Fig 3:**
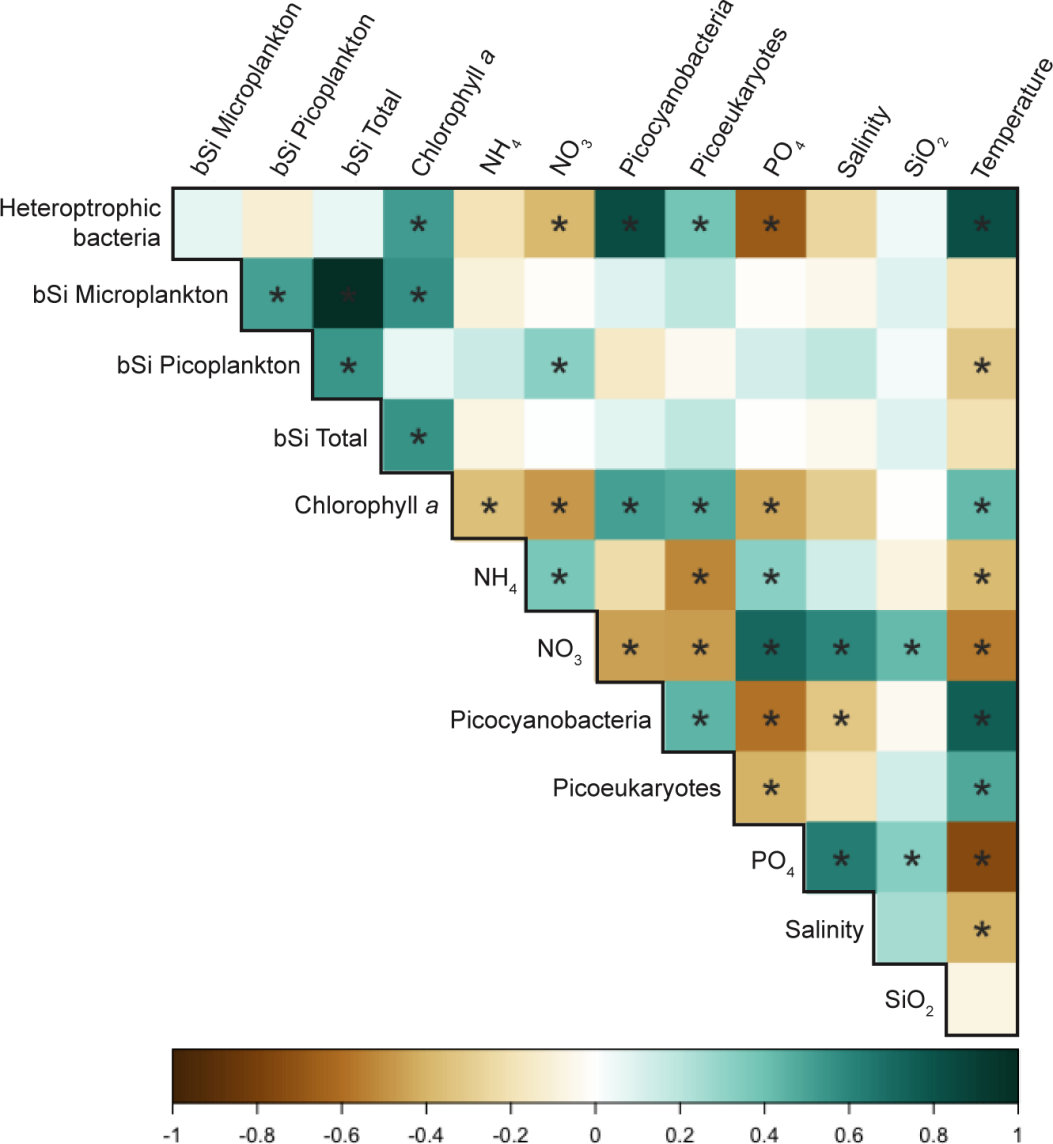
Spearman correlations for LMO samples using environmental and biological data from 41 LMO samplings. The factors included are picoplankton; microplankton; total bSi standing stock; total chlorophyll *a*; log-transformed flow cytometry cell counts of heterotrophic bacteria, PPE, and picocyanobacteria; nutrient concentrations of NH_4_, NO_3_, PO_4_, and SiO_2_; salinity; and temperature. An asterisk (*) in the square indicates a statistically significant (*P* ≤ 0.05) relationship.

A Spearman correlation analysis was done to identify relationships between the various environmental and biological factors measured at LMO (Fig. 3). Picoplankton and microplankton bSi were positively correlated to each other, and both correlated positively with total bSi. There was a significant positive correlation between microplankton bSi and total chlorophyll *a*. NH_4_ had the most significant positive correlation with picoplankton bSi, while temperature had a significant negative correlation. Temperature had significant positive correlations with total chlorophyll *a* and picoplankton abundances (heterotrophic bacteria, picoeukaryotes, and picocyanobacteria) and negative correlations with several environmental factors (salinity, PO_4_, and NO_3_). Salinity had significant positive correlations with NO_3_ and PO_4_.

### Initial community composition and nutrient addition response in the microcosms

**Fig 4:**
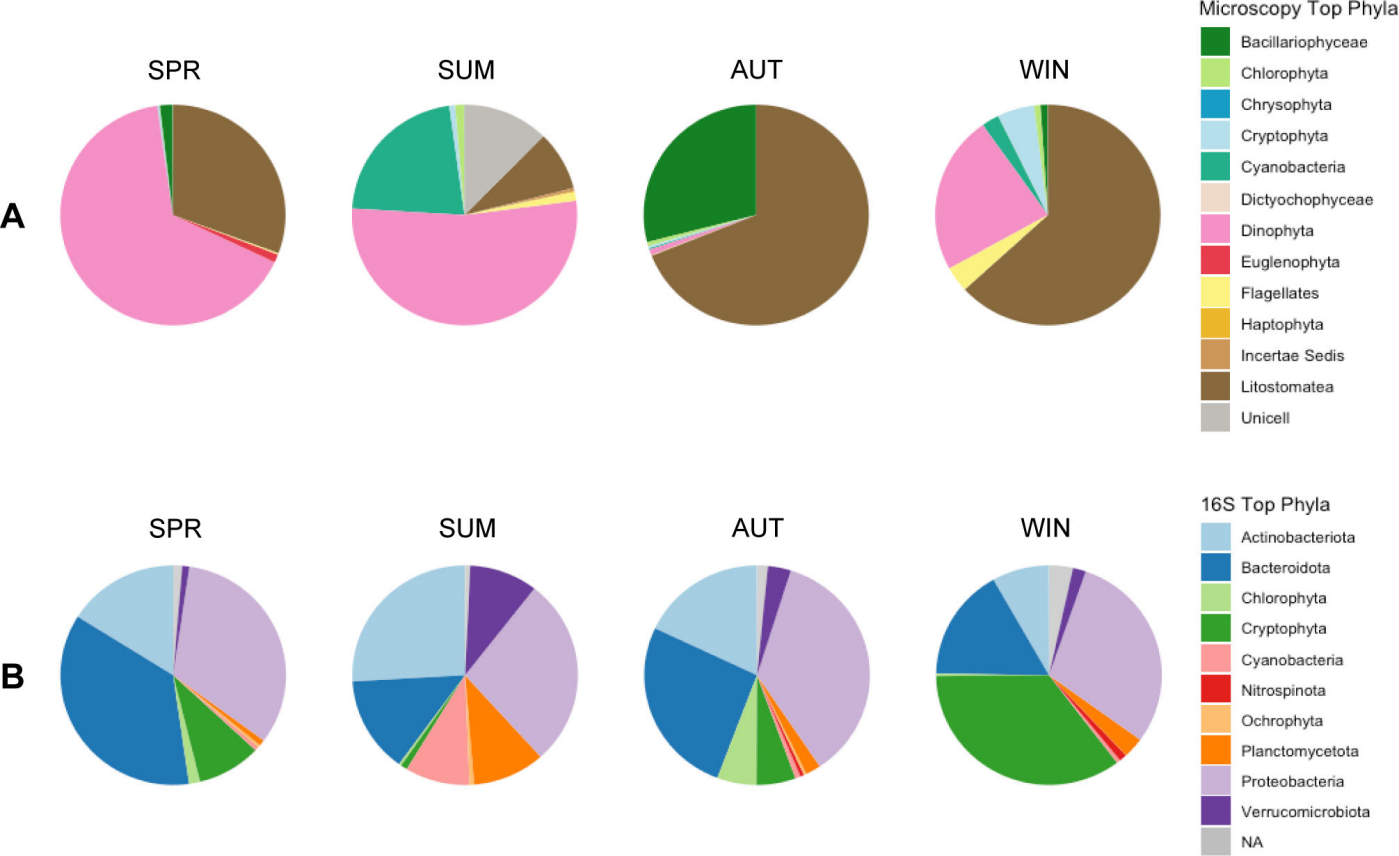
Major taxonomic groups at the initial time point (t0) of the four microcosm experiments (SPR, SUM, AUT, and WIN) of phytoplankton identified microscopically (>5 µm) (**A**) and picoplankton (0.22–3 µm) identified using 16S rRNA gene amplicons (**B**). The picoplankton proportions are the mean relative abundance of sequences from five replicates. The phytoplankton carbon biomass proportions are calculated from single microscopy samples.

Microscopy and 16S rRNA gene data confirmed that community composition of both the picoplankton and microplankton was distinct between microcosm experiments at the initial time point (t0; Fig. 4). This follows what has been previously observed at LMO during different seasons (27, 65). Within the larger phytoplankton community, in SPR and SUM, *Dinophyta* dominated (proportion of carbon biomass 53%–66%), while in AUT and WIN, *Litostomatea* made up the majority (proportion of carbon biomass 63%–69%) of carbon biomass (Fig. 4A). In SPR, *Litostomatea* (proportion of carbon biomass 31%) contributed to a significant proportion of the total biomass. *Cyanobacteria* (proportion of carbon biomass 22%) and *Bacillariophyceae* (diatoms; proportion of carbon biomass 29%) contributed in higher proportions to SUM and AUT communities, respectively. There were no obvious community composition differences related to treatment at the final time point (PcoA analysis in Materials and Methods; data not shown). Instead, general trends included increases in the proportion of *Dinophyta* in SPR, *Cyanobacteria* in SUM, and diatoms in AUT (Fig. S2). In SPR, SUM, and AUT, the picoplankton community was composed primarily of *Actinobacteria, Bacteroidota,* and *Proteobacteria* (total relative abundance 68%–85%), while in WIN, their contribution was lower (total relative abundance 54%) (Fig. 4B). The SUM community was distinct for its higher proportion of *Cyanobacteria* and *Planctomycetota* (relative abundance 9% and 10%, respectively), and the WIN community was distinct for its high proportion of *Cryptophyta* (relative abundance 35%). At the final time point, there did not seem to be any community composition differences between samples with different nutrient additions (PCA analysis in Materials and Methods; data not shown), though t0 and t65 samples had distinct communities (Fig. S3).

### Microbial response to nutrient enrichment in microcosms

**Fig 5:**
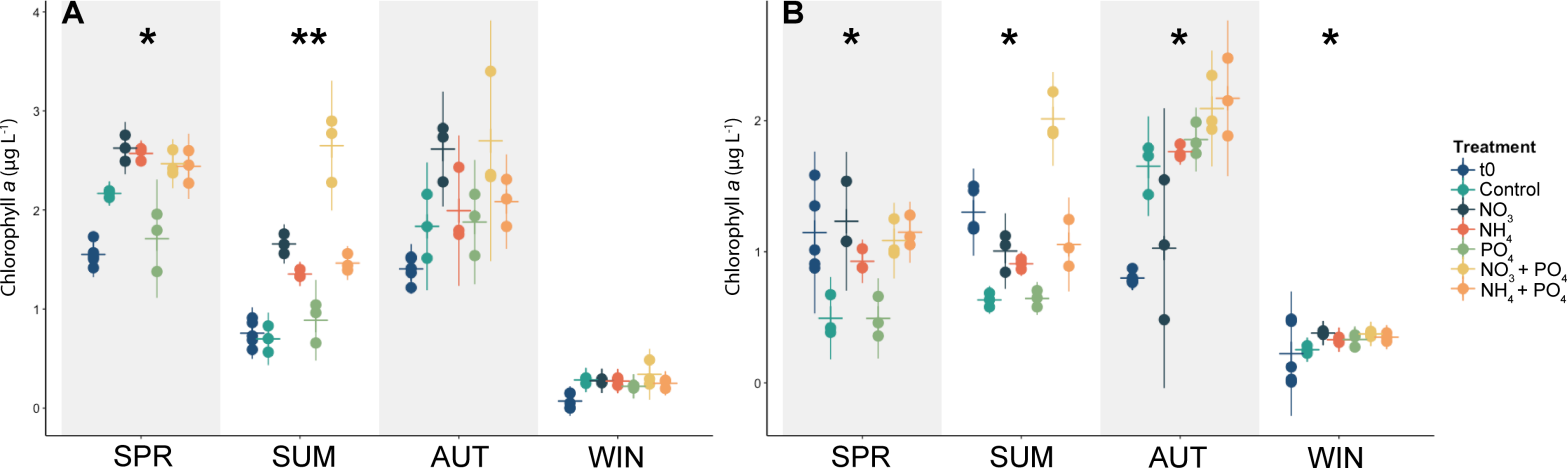
Chlorophyll *a* concentration in microplankton (**A**) and picoplankton (**B**) over the course of the microcosm experiments from the initial (t0, dark green) to the final (t65, lighter green shades) time point. Note the differences in values for the *y* axes. Asterisk(s) indicate significant *P* values (Kruskal-Wallis ANOVA, *P ≤ 0.05 and **P ≤ 0.01).

Nutrient enrichment had differing effects on chlorophyll *a* concentration in the different microcosm experiments (Fig. 5). In the microplankton fraction, differences were significant in SPR and SUM (Kruskal-Wallis ANOVA, *P* < 0.05) but not in AUT or WIN (Table S2). Dunn’s *post hoc* test revealed significant differences in the SUM microcosm between the control and NO_3_ treatment (*P* = 0.008). Chlorophyll *a* concentrations in the picoplankton fraction differed significantly in all microcosm experiments (Kruskal-Wallis ANOVA, *P* < 0.05; Table S2). Dunn’s *post hoc* test showed significant differences between the control and NO_3_ + PO_4_ treatment in the SUM (*P* = 0.011) and the control and NO_3_ treatment in the WIN microcosm (*P* = 0.024). Nitrogen (as either NO_3_ or NH_4_) was the primary limiting nutrient for both microplankton and picoplankton, which was most evident in the SPR and SUM microcosms.

### Nutrient enrichment impact on bSi and dSi in microcosms

**Fig 6:**
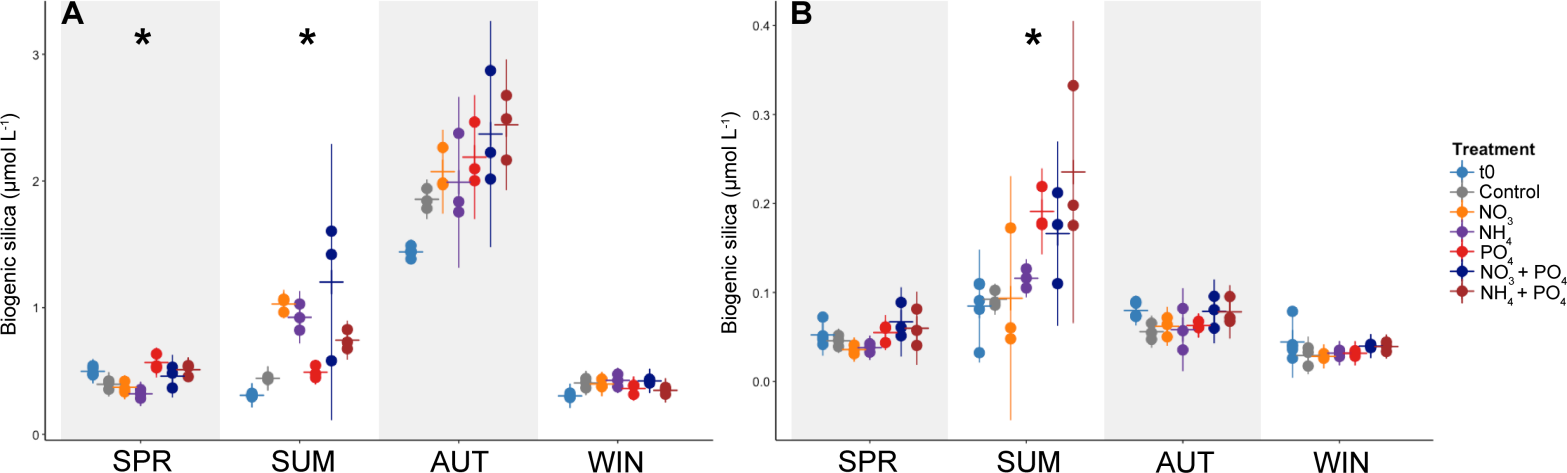
Biogenic silica concentrations in microplankton (**A**) and picoplankton (**B**) over the course of the microcosm experiment from the initial (t0, black) to the final (t65, gray, blue, and purple shades) time point. Note the differences in values for the *y* axes. Asterisk indicates significant *P* values (Kruskal-Wallis ANOVA, *P ≤ 0.05).

Biogenic Si concentrations in the microplankton (Fig. 6A) and picoplankton (Fig. 6B) fractions changed over time, from the initial to the final time point and between different treatments. In the microplankton, these differences were statistically significant in SPR and SUM (Kruskal-Wallis ANOVA, *P* < 0.05), though not in AUT or WIN (Table S3). Further analysis with Dunn’s *post hoc* test showed differences in bSi between the control and NO_3_ treatment (*P* = 0.035) in the SUM microcosm. However, picoplankton bSi differed significantly only in SUM (*P* < 0.05; Table S3), and a Dunn’s *post hoc* test did not show significant differences between the control and treatment groups. At the final time point (t65), dSi concentrations in the nutrient-enriched microcosms (7.1–18.7 µmol L^−1^) were similar to the controls, indicating that significant dSi drawdown did not occur (Fig. S4).

### Biological and environmental factors correlating with bSi in microcosms

**Fig 7:**
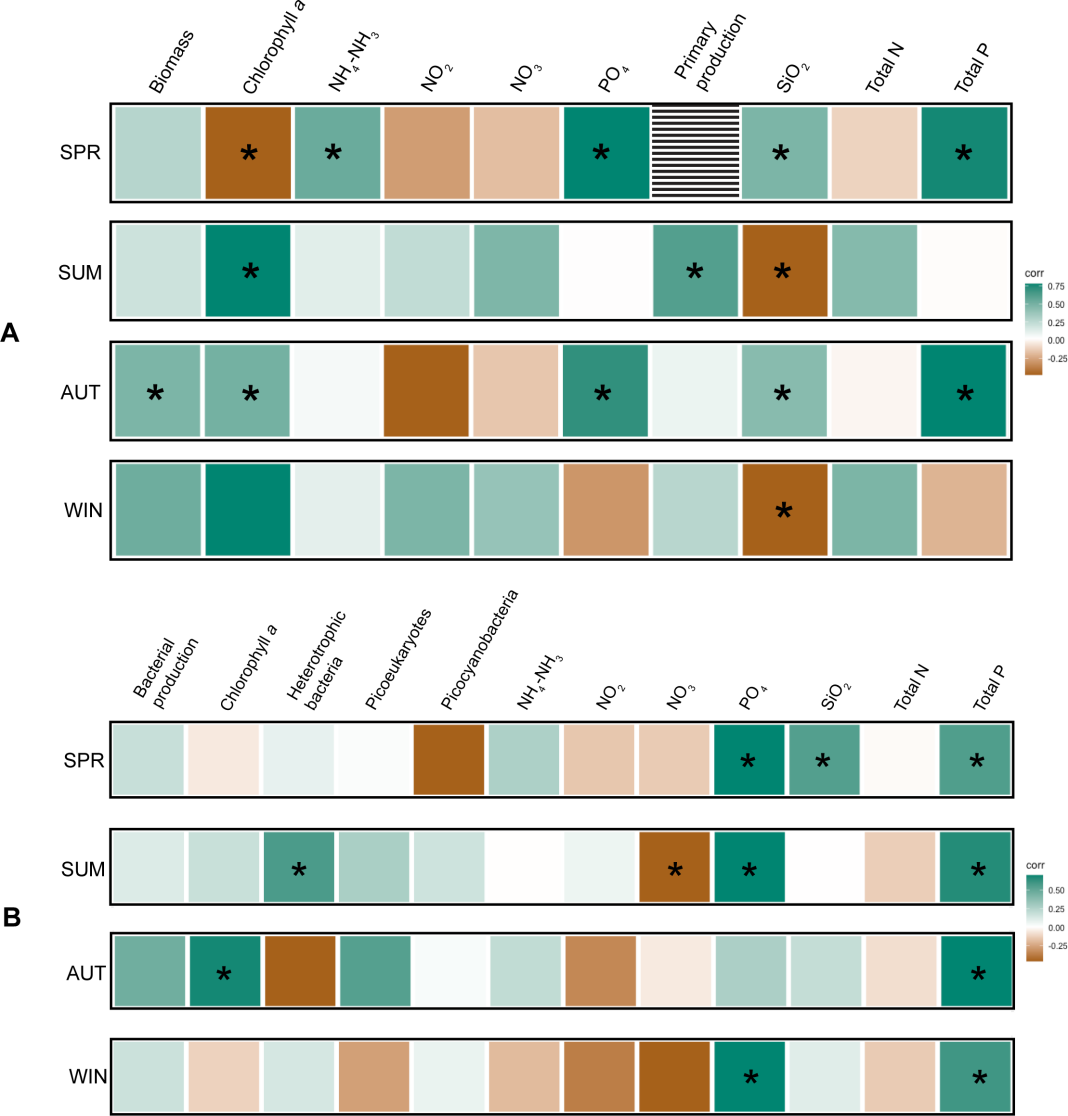
Spearman correlations for the microplankton (**A**) and picoplankton (**B**), correlating bSi to selected environmental and biological factors measured at the final time point (t65) of the microcosm experiments (SPR, SUM, AUT, and WIN). The environmental and biological factors for the microplankton (**A**) are total carbon biomass; microplankton chlorophyll *a*; nutrient concentrations of NH_4_-NH_3_, NO_2_, NO_3_, PO_4_, SiO_2_, total N, total P; and primary production. The environmental and biological factors for the picoplankton (**B**) are bacterial production picoplankton chlorophyll *a*; log-transformed flow cytometry cell counts of heterotrophic bacteria, photosynthetic picoeukaryotes, and picocyanobacteria; nutrient concentrations of NH_4_-NH_3_, NO_2_, NO_3_, PO_4_, SiO_2_, total N, and total P. Data from the t65 control and treatment replicates (*n* = 18) were compiled together for each microcosm. An asterisk (*) in the square indicates a statistically significant (*P* ≤ 0.05) relationship. Note that primary production data were not collected for microcosm SPR.

Spearman correlations on the data collected from the microcosm experiments showed that, in microplankton, there were strong positive correlations between bSi and chlorophyll *a* concentrations in SUM, AUT, and WIN, which was also seen in the LMO data set (Fig. 7A). Carbon biomass data of the different phytoplankton classes identified through microscopy revealed that diatoms were the only organismal group significantly positively correlated with microplankton bSi in microcosm experiments (Fig. S5). In SPR and AUT, positive correlations between microplankton bSi with environmental variables PO_4_, total P, and dSi were also observed. In contrast to the LMO data set, picoplankton bSi concentrations did not positively correlate to NH_4_-NH_3_ concentrations in any of the experiments. Instead, strong positive correlations between picoplankton bSi and P (PO_4_ and total P) were observed across all microcosms (Fig. 7B), though in AUT the correlation with PO_4_ was not statistically significant. In contrast, positive correlations with biological variables were inconsistent between experiments. In SUM, there was a strong correlation with heterotrophic bacterial abundances, while in AUT, with picoplankton chlorophyll *a*.

## DISCUSSION

Changes in Si are dependent on different biological and physical parameters, and these changes are visible at different timescales. Generally, long-term studies of bSi standing stock and production dynamics are rare in the literature (17). Studies that incorporate size fractionation, more so. At the long-term Bermuda Atlantic Time Series site in the oligotrophic North Atlantic subtropical gyre, bSi variability was visible at several different timescales: short-term, seasonal, and interannual (17). Long-term decreases in bSi standing stock and production were linked to decreases in diatom abundances and increases in dinoflagellates and prasinophytes. Short-term changes to bSi were attributed to meteorological events like storms or mesoscale eddies (17). In the dSi-replete central Baltic Sea, we were able to examine bSi standing stock in two size fractions at short-term and seasonal timescales (incorporating 2 years). We found that the majority of bSi in surface waters was associated with microplankton, corresponding primarily to changes in the composition and biomass of phytoplankton. However, it is notable that picoplankton, on several occasions, comprised up to 10%–20% of bSi. With the consideration that a fraction of the bSi measured may be due to dissolved LSi or cell debris, we still see that picoplankton make up a significant portion of the bSi stock throughout different seasons. Using long-term sampling, we were able to observe the fluctuations in both microplankton and picoplankton bSi concentrations.

In the microcosm experiments, there was strong evidence that diatoms drove bSi accumulation. In AUT, there were proportionally large diatom populations, and we measured the highest bSi concentrations of the four microcosm experiments (t65: 1.76–2.87 µmol L^−1^). In the SUM microcosms, there were several treatments where bSi concentrations and diatom biomass both increased in comparison to controls. Furthermore, in the SUM and AUT microcosms, there were significant positive correlations between diatom biomass and bSi. Contrary to this, a negative correlation between microplankton bSi and chlorophyll *a* was observed in SPR. In SPR, instead of diatoms, the phylum *Dinophyta*, commonly referred to as dinoflagellates, dominated carbon biomass. Dinoflagellates are not thought to be prominent players in silica cycling, though select dinoflagellate species have silicon ion transporter-like sequences in their genomes (9). Overall, these observations emphasize the important role diatoms have in moderating bSi dynamics in the Baltic Sea. This is particularly of interest due to the observed shifts from diatom-dominated spring blooms to ones dominated by dinoflagellates (34–36), as well as evidence for more intense autumn diatom blooms (66). Direct consequences of these changes can include absent diatom blooms giving other bSi-producing organisms undisturbed access to dSi in the spring, and the Baltic Proper becoming an area where diatom growth is limited by dSi (as well as nitrogen) availability in the autumn. In light of the importance of diatoms to silica cycling, the effects of their community changes on silica cycling should be followed.

The range of the contribution of picoplankton to bSi stock in the Baltic Sea (1.1%–21.6%) fell within the broad range observed in diverse oligotrophic environments (11–16). Notably, this range was seen over 2 years of biweekly observations at one sampling point, while previous studies were conducted over multiple sampling locations and depths. Our data indicated a positive correlation with NH_4_ concentrations, but no correlations with picoplankton cell abundances were observed. This differs from correlations observed in short-term field research cruise campaigns conducted in oligotrophic environments, where positive correlations between bSi in the <2 µm fraction with temperature (14, 16), light (16), *Synechococcus* cell abundance, *Prochlorococcus* cell abundance (14, 16), and PPE cell abundance (15) have been reported. We observed seasonal changes in picoplankton bSi concentrations; they peaked in autumn and winter (approximately 0.05–0.12 µmol L^−1^ compared to 0.01–0.03 µmol L^−1^ in spring and summer), though the contribution to total bSi was lower (approximately 1%–10% compared to 6%–21% in spring and summer) due to co-occurring high microplankton bSi concentrations. The large range in bSi over a yearly cycle at one sampling point emphasizes the importance of studying different environments and including timescale and spatial factors when assessing bSi accumulation in picoplankton.

In our microcosm experiments, particularly in SUM, phosphorus additions seemed to promote bSi accumulation in picoplankton. Laboratory studies with *Synechococcus* isolates have been used to probe the connection between phosphorus and dSi uptake. Previous studies have shown that *Synechococcus* strains acclimated to phosphorus-limited media conditions have higher bSi concentrations relative to controls (67). Other strains grown without phosphorus in the media also showed higher bSi accumulation rates, and phosphorus additions resulted in lower bSi accumulation rates (5). However, some of these strains, when grown in high dSi, low phosphorus (10:1 and 5:1 ratio) conditions for more than 10 generations, had lower bSi concentrations than cells acclimated to a 1:1 ratio dSi and phosphorus. Though the Baltic Proper is not, as far as we are aware, phosphorus limited, we see similar patterns in our microcosm experiments. Phosphorus concentrations are positively correlated with bSi concentrations in picoplankton for all seasons, showing that phosphorus influences bSi accumulation. Our experiments suggest that in environments where phosphorus inputs stimulate growth, one can expect higher bSi concentrations in picoplankton, giving a direction for future studies on bSi accumulation.

Diverse picoplankton associated with the smaller size fraction could have had a role in bSi accumulation in this study. 16S rRNA gene plastid sequences assigned to known biosilicifiers, including diatoms primarily of the class Coscinodiscophyceae and Dictyochophyceae (silicoflagellates), were found in all microcosm samples. However, the relative abundance of the phylum Ochrophyta, to which Coscinodiscophyceae and Dictyochophyceae fall under, was low in comparison to other phyla detected in the pico-sized fraction. There were a few positive correlations between picoplankton abundance and biomass with bSi in the microcosm experiments. We saw a significant correlation with heterotrophic bacterial cell abundances in SUM and with chlorophyll *a* in AUT. Additionally, in AUT, there was a strong but not significant negative correlation with heterotrophic bacterial cell abundances and a positive correlation with picoeukaryotic cell abundances. In natural marine environments, bSi quotas in individual *Synechococcus* cells are known to be highly variable (4, 13). This would potentially preclude a linear relationship between picoplankton bSi, chlorophyll *a,* and cell abundances. While we know that some picoeukaryote (6) and *Synechococcus* (37) isolates from the Baltic Proper are capable of accumulating bSi, we do not know the distribution of bSi in individual cells. Moreover, we cannot confirm that all strains can accumulate bSi. Another unknown is heterotrophic bacteria, which comprise the biggest portion (i.e., ratio of cell abundances) of the picoplankton at LMO. Biosilicifying bacteria from geothermal environments have been investigated for their utilization of silica as a protective coating (68, 69), and there is recent evidence that there is intracellular uptake in ubiquitous soil bacteria *Bacillus cereus* (70). Biosilicifying bacteria from the genus *Bacillus*, and potentially others, are theorized to contribute to the terrestrial silica cycle (71). However, as far as we are aware, there is no research on silica accumulation capabilities in heterotrophic bacteria in marine environments, nor is their potential contribution estimated. Additionally, as size fractionation is only a technical separation of biomass, we cannot exclude the possibility of debris from larger cells being collected on the pico fraction filter.

The method we used in our study may have influenced the results of the bSi measurements. One limitation of the NaOH extraction method used in this study to measure what we refer to as bSi standing stock is that the measured end product cannot be determined to be purely of biotic (biogenic) origin. It has been observed that LSi can be digested alongside bSi during the NaOH extraction (72). Additionally, detritus from larger cells passing through 3 µm filters has been theorized to make up a significant part of what is measured as bSi (11, 73). LSi measurements of select samples show that it made up 5.9%–18% of total particulate (bSi + LSi) Si (Table S1). Therefore, the influence of LSi on bSi measurements, in this case, appears minimal. Detrital enrichment of bSi in the pico-sized fraction has occurred over a period of weeks in a previous study (74), while our microcosm experiments lasted only 65 hours (2.75 days). The limited timeframe of this study helps ensure that the bSi measured at the final time point of the experiments was a measure of biotic enrichment and not detrital, though this should have been validated with microscopic methods. In the future, methods that measure bSi uptake and production, such as Si isotope fractionation, as well as single-cell methods like synchrotron-based X-ray fluorescence microscopy and scanning electron microscopy (SEM), are likely to provide novel knowledge in addition to the NaOH extraction method for a more holistic understanding of silica cycling.

This study offers both new insights into silica cycling in the Baltic Sea and bSi accumulation in natural communities. Consistent with other aquatic environments, we find that diatoms are principal contributors to bSi, with concentration shifts linked to changes in their biomass and composition. Meanwhile, the proportional contribution of picoplankton is, at times, significant. We propose that silica cycling in the Baltic Sea is undergoing change due to the shifts in spring and autumn phytoplankton communities. Additionally, we speculate that climate change-associated increases in picoplankton abundances and contribution to plankton biomass will likely magnify the contribution of picoplankton to silica cycling in this environment. Phosphorus-induced stimulation of growth and bSi accumulation indicates a potential avenue for bSi uptake in picoplankton. Future targeted studies on molecular mechanisms involved in phosphorus and bSi accumulation in natural communities can help resolve the relationship between phosphorus and bSi availability in picoplankton. In addition, we suggest that future studies investigate bSi on multiple timescales. This is relevant not only for understanding the mechanisms of bSi accumulation but also for integrating interactions between microorganisms and studying their contribution to the silica cycle.

## Data Availability

The amplicon data have been submitted to the European Nucleotide Archive (ENA) with the accession number PRJEB82697.

## AFTER EPUB

*[This article was published on 28 April 2025 with a portion of Table 1 missing. The table was corrected in the current version, posted on 30 April 2025.]*

## SUPPLEMENTAL MATERIAL

Supplemental material aem.00676-25-s0001.docx Figures S1 to S5; Tables S1 to S3.

ASM does not own the copyrights to Supplemental Material that may be linked to, or accessed through, an article. The authors have granted ASM a non-exclusive, world-wide license to publish the Supplemental Material files. Please contact the corresponding author directly for reuse.

## References

[1] Tréguer PJ, Sutton JN, Brzezinski M, Charette MA, Devries T, Dutkiewicz S, Ehlert C, Hawkings J, Leynaert A, Liu SM, Llopis Monferrer N, López-Acosta M, Maldonado M, Rahman S, Ran L, Rouxel O. 2021. Reviews and syntheses: the biogeochemical cycle of silicon in the modern ocean. Biogeosciences 18:1269–1289. doi:10.5194/bg-18-1269-2021

[2] Llopis Monferrer N, Boltovskoy D, Tréguer P, Sandin MM, Not F, Leynaert A. 2020. Estimating biogenic silica production of rhizaria in the global ocean. Global Biogeochem Cycles 34:E2019GB006286. doi:10.1029/2019GB006286

[3] Laget M, Drago L, Panaïotis T, Kiko R, Stemmann L, Rogge A, Llopis-Monferrer N, Leynaert A, Irisson J-O, Biard T. 2024. Global census of the significance of giant mesopelagic protists to the marine carbon and silicon cycles. Nat Commun 15:3341. doi:10.1038/s41467-024-47651-4 38684684 PMC11058905

[4] Baines SB, Twining BS, Brzezinski MA, Krause JW, Vogt S, Assael D, McDaniel H. 2012. Significant silicon accumulation by marine picocyanobacteria. Nature Geosci 5:886–891. doi:10.1038/ngeo1641

[5] Brzezinski MA, Krause JW, Baines SB, Collier JL, Ohnemus DC, Twining BS. 2017. Patterns and regulation of silicon accumulation in Synechococcus spp. J Phycol 53:746–761. doi:10.1111/jpy.12545 28457002

[6] Churakova Y, Aguilera A, Charalampous E, Conley DJ, Lundin D, Pinhassi J, Farnelid H. 2023. Biogenic silica accumulation in picoeukaryotes: novel players in the marine silica cycle. Environ Microbiol Rep 15:282–290. doi:10.1111/1758-2229.13144 36992638 PMC10316375

[7] Durak GM, Taylor AR, Walker CE, Probert I, de Vargas C, Audic S, Schroeder D, Brownlee C, Wheeler GL. 2016. A role for diatom-like silicon transporters in calcifying coccolithophores. Nat Commun 7:10543. doi:10.1038/ncomms10543 26842659 PMC4742977

[8] Tostevin R, Snow JT, Zhang Q, Tosca NJ, Rickaby REM. 2021. The influence of elevated SiO_2_ (aq) on intracellular silica uptake and microbial metabolism. Geobiology 19:421–433. doi:10.1111/gbi.12442 33838079

[9] Marron AO, Ratcliffe S, Wheeler GL, Goldstein RE, King N, Not F, de Vargas C, Richter DJ. 2016. The evolution of silicon transport in eukaryotes. Mol Biol Evol 33:3226–3248. doi:10.1093/molbev/msw209 27729397 PMC5100055

[10] Brzezinski MA, Varela DE, Jenkins BD, Buck KN, Kafrissen SM, Jones JL. 2022. The upper ocean silicon cycle of the subarctic Pacific during the EXPORTS field campaign. Elementa (Wash D C) 10. doi:10.1525/elementa.2021.00087

[11] Krause JW, Brzezinski MA, Baines SB, Collier JL, Twining BS, Ohnemus DC. 2017. Picoplankton contribution to biogenic silica stocks and production rates in the Sargasso Sea. Global Biogeochem Cycles 31:762–774. doi:10.1002/2017GB005619

[12] Leblanc K, Cornet V, Rimmelin-Maury P, Grosso O, Hélias-Nunige S, Brunet C, Claustre H, Ras J, Leblond N, Quéguiner B. 2018. Silicon cycle in the tropical South pacific: contribution to the global si cycle and evidence for an active pico-sized siliceous plankton. Biogeosciences 15:5595–5620. doi:10.5194/bg-15-5595-2018

[13] Ohnemus DC, Rauschenberg S, Krause JW, Brzezinski MA, Collier JL, Geraci-Yee S, Baines SB, Twining BS. 2016. Silicon content of individual cells of Synechococcus from the North Atlantic Ocean. Mar Chem 187:16–24. doi:10.1016/j.marchem.2016.10.003

[14] Wei Y, Sun J, Chen Z, Zhang Z, Zhang G, Liu X. 2021. Significant contribution of picoplankton size fraction to biogenic silica standing stocks in the Western Pacific Ocean. Prog Oceanogr 192:102516. doi:10.1016/j.pocean.2021.102516

[15] Wei Y, Wang X, Gui J, Sun J. 2021. Significant pico- and nanoplankton contributions to biogenic silica standing stocks and production rates in the oligotrophic eastern indian ocean. Ecosystems (N Y, Print) 24:1654–1669. doi:10.1007/s10021-021-00608-w

[16] Wei Y, Zhang Z, Cui Z, Sun J. 2021. Size-fractionated biogenic silica standing stocks and carbon biomass in the western tropical north pacific: evidence for the ecological importance of pico-sized plankton in oligotrophic gyres. Front Mar Sci 8:691367. doi:10.3389/fmars.2021.691367

[17] Krause JW, Lomas MW, Nelson DM. 2009. Biogenic silica at the bermuda atlantic time‐series study site in the sargasso sea: temporal changes and their inferred controls based on a 15‐year record. Global Biogeochem Cycles 23:GB3004. doi:10.1029/2008GB003236

[18] Granéli E, Wallström K, Larsson U, Granéli W, Elmgren R. 1990. Nutrient Limitation of Primary Production in the Baltic Sea Area. Marine Eutrophication 19:142–151.

[19] Wasmund N, Nausch G, Feistel R. 2013. Silicate consumption: an indicator for long-term trends in spring diatom development in the Baltic Sea. J Plankton Res 35:393–406. doi:10.1093/plankt/fbs101

[20] Danielsson Å, Papush L, Rahm L. 2008. Alterations in nutrient limitations — Scenarios of a changing Baltic Sea. J Mar Syst 73:263–283. doi:10.1016/j.jmarsys.2007.10.015

[21] Swedish county administration boards, Swedish municipalities, Swedish coalitions of water conservation, Swedish Meteorological and Hydrological Institute. 2023

[22] Carman R, Aigars J. 1997. Vertical and spatial distribution of biogenic silica in the sediment of the gulf of Riga, Baltic Sea. Toxicol Environ Chem 60:245–259. doi:10.1080/02772249709358468

[23] Pastuszak M, Conley DJ, Humborg C, Witek Z, Sitek S. 2008. Silicon dynamics in the Oder estuary, Baltic Sea. J Mar Syst 73:250–262. doi:10.1016/j.jmarsys.2007.10.013

[24] Conley DJ, Humborg C, Smedberg E, Rahm L, Papush L, Danielsson Å, Clarke A, Pastuszak M, Aigars J, Ciuffa D, Mörth C-M. 2008. Past, present and future state of the biogeochemical Si cycle in the Baltic Sea. J Mar Syst 73:338–346.

[25] Tallberg P, Räike A, Lukkari K, Leivuori M, Lehtoranta J, Pitkänen H. 2012. Horizontal and vertical distribution of biogenic silica in coastal and profundal sediments of the Gulf of Finland (northeastern Baltic Sea). Boreal Environ Res 17:347–362.

[26] Bunse C, Israelsson S, Baltar F, Bertos-Fortis M, Fridolfsson E, Legrand C, Lindehoff E, Lindh MV, Martínez-García S, Pinhassi J. 2018. High frequency multi-year variability in baltic sea microbial plankton stocks and activities. Front Microbiol 9:3296. doi:10.3389/fmicb.2018.03296 30705671 PMC6345115

[27] Fridolfsson E, Bunse C, Lindehoff E, Farnelid H, Pontiller B, Bergström K, Pinhassi J, Legrand C, Hylander S. 2023. Multiyear analysis uncovers coordinated seasonality in stocks and composition of the planktonic food web in the Baltic Sea proper. Sci Rep 13:11865. doi:10.1038/s41598-023-38816-0 37481661 PMC10363133

[28] Sondergaard M, Jensen L, Ærtebjerg G. 1991. Picoalgae in Danish coastal waters during summer stratification. Mar Ecol Prog Ser 79:139–149. doi:10.3354/meps079139

[29] Stal L, Staal M, Villbrandt M. 1999. Nutrient control of cyanobacterial blooms in the Baltic Sea. Aquat Microb Ecol 18:165–173. doi:10.3354/ame018165

[30] Ohlendieck U, Stuhr A, Siegmund H. 2000. Nitrogen fixation by diazotrophic cyanobacteria in the Baltic Sea and transfer of the newly fixed nitrogen to picoplankton organisms. J Mar Syst 25:213–219. doi:10.1016/S0924-7963(00)00016-6

[31] Tamm M, Laas P, Freiberg R, Nõges P, Nõges T. 2018. Parallel assessment of marine autotrophic picoplankton using flow cytometry and chemotaxonomy. Sci Total Environ 625:185–193. doi:10.1016/j.scitotenv.2017.12.234 29289004

[32] Zufia JA, Legrand C, Farnelid H. 2022. Seasonal dynamics in picocyanobacterial abundance and clade composition at coastal and offshore stations in the Baltic Sea. Sci Rep 12:14330. doi:10.1038/s41598-022-18454-8 35995823 PMC9395346

[33] Legrand C, Fridolfsson E, Bertos-Fortis M, Lindehoff E, Larsson P, Pinhassi J, Andersson A. 2015. Interannual variability of phyto-bacterioplankton biomass and production in coastal and offshore waters of the Baltic Sea. Ambio 44:427–438. doi:10.1007/s13280-015-0662-8 26022325 PMC4447688

[34] Klais R, Tamminen T, Kremp A, Spilling K, Olli K. 2011. Decadal-scale changes of dinoflagellates and diatoms in the anomalous baltic sea spring bloom. PLoS One 6:e21567. doi:10.1371/journal.pone.0021567 21747911 PMC3126836

[35] Klais R, Tamminen T, Kremp A, Spilling K, An BW, Hajdu S, Olli K. 2013. Spring phytoplankton communities shaped by interannual weather variability and dispersal limitation: mechanisms of climate change effects on key coastal primary producers. Limnology & Oceanography 58:753–762. doi:10.4319/lo.2013.58.2.0753

[36] Spilling K, Olli K, Lehtoranta J, Kremp A, Tedesco L, Tamelander T, Klais R, Peltonen H, Tamminen T. 2018. Shifting diatom—dinoflagellate dominance during spring bloom in the baltic sea and its potential effects on biogeochemical cycling. Front Mar Sci 5:327. doi:10.3389/fmars.2018.00327

[37] Aguilera A, Lundin D, Charalampous E, Churakova Y, Tellgren-Roth C, Śliwińska-Wilczewska S, Conley DJ, Farnelid H, Pinhassi J. n.d. The evaluation of biogenic silica in brackish and freshwater strains reveals links between phylogeny and silica accumulation in picocyanobacteria. Appl Environ Microbiol 0:e02527–24. doi:10.1128/aem.02527-24 PMC12016540 40145754

[38] Valderrama JC. 1995. Methods of nutrient analysis. InG. M. HallagraeffAnderson DM, Cembella AD (ed), Manual on Harmful Marine Microalgae. IOC Manuals and Guides), Mumbai.

[39] Hansen HP, Koroleff F. 1999. Determination of nutrients. In Wiley VCH, Grasshoff K, Kremling K, Ehrhardt M (ed), Methods of Seawater Analysis. Weinheim, Germany.

[40] Krause JW, Brzezinski MA, Siegel DA, Thunell RC. 2013. Biogenic silica standing stock and export in the Santa Barbara Channel ecosystem. JGR Oceans 118:736–749. doi:10.1029/2012JC008070

[41] Brzezinski MA, Nelson DM. 1995. The annual silica cycle in the Sargasso Sea near Bermuda. Deep Sea Research Part I: Oceanographic Research Papers 42:1215–1237. doi:10.1016/0967-0637(95)93592-3

[42] Dürr HH, Meybeck M, Hartmann J, Laruelle GG, Roubeix V. 2011. Global spatial distribution of natural riverine silica inputs to the coastal zone. Biogeosciences 8:597–620. doi:10.5194/bg-8-597-2011

[43] Tréguer PJ, De La Rocha CL. 2013. The world ocean silica cycle. Ann Rev Mar Sci 5:477–501. doi:10.1146/annurev-marine-121211-172346 22809182

[44] Leblanc K, Quéguiner B, Garcia N, Rimmelin P, Raimbault P. 2003. Silicon cycle in the NW Mediterranean Sea: seasonal study of a coastal oligotrophic site. Oceanologica Acta 26:339–355. doi:10.1016/S0399-1784(03)00035-5

[45] Jespersen AM, Christoffersen K. 1987. Measurements of chlorophyll-a from phytoplankton using ethanol as extraction solvent. archiv_hydrobiologie 109:445–454. doi:10.1127/archiv-hydrobiol/109/1987/445

[46] Alegria Zufia J, Farnelid H, Legrand C. 2021. Seasonality of Coastal Picophytoplankton Growth, Nutrient Limitation, and Biomass Contribution. Front Microbiol 12:786590. doi:10.3389/fmicb.2021.786590 34938282 PMC8685431

[47] Gasol JM, Del Giorgio PA. 2000. Using flow cytometry for counting natural planktonic bacteria and understanding the structure of planktonic bacterial communities. Sci mar 64:197–224. doi:10.3989/scimar.2000.64n2197

[48] Gargas E. 1975. A manual for phytoplankton primary production studies in the Baltic. Baltic Marine Biologists:Water Quality Institute, Hørsholm, Denmark.

[49] Smith DC, Azam F. 1992. A simple, economical method for measuring bacterial protein synthesis rates in seawater using 3H-leucine. Mar Microb Food Webs 6:107–114.

[50] Gasol JM, Doval MD, Pinhassi J, Calderón-Paz JI, Guixa-Boixareu N, Vaqué D, Pedrós-Alió C. 1998. Diel variations in bacterial heterotrophic activity and growth in the northwestern Mediterranean Sea. Mar Ecol Prog Ser 164:107–124. doi:10.3354/meps164107

[51] Simon M, Azam F. 1989. Protein content and protein synthesis rates of planktonic marine bacteria. Mar Ecol Prog Ser 51:201–213. doi:10.3354/meps051201

[52] Olenina I, Hajdu S, Edler L, Andersson A, Wasmund N, Busch S, Gromisz S, Huseby S, Huttunen M, Jaanus A, Kokkonen P, Ledaine I, Niemkiewicz E. 2006. Biovolumes and size-classes of phytoplankton in the Baltic Sea. HELCOM BaltSea Environ Proc No 106

[53] Edler L. 1979. Recommendations on methods for marine biological studies in the Baltic Sea: phytoplankton and chlorophyll*.* University of Stockholm, Stockholm.

[54] HELCOM Phytoplankton Expert Group. 2013. Phytoplankton biovolume and carbon content*.* Available from: https://www.ices.dk/data/Documents/ENV/PEG_BVOL.zip

[55] Herlemann DP, Labrenz M, Jürgens K, Bertilsson S, Waniek JJ, Andersson AF. 2011. Transitions in bacterial communities along the 2000 km salinity gradient of the Baltic Sea. ISME J 5:1571–1579. doi:10.1038/ismej.2011.41 21472016 PMC3176514

[56] Straub D, Blackwell N, Langarica-Fuentes A, Peltzer A, Nahnsen S, Kleindienst S. 2020. Interpretations of environmental microbial community studies are biased by the selected 16S rRNA (gene) amplicon sequencing pipeline. Front Microbiol 11:550420. doi:10.3389/fmicb.2020.550420 33193131 PMC7645116

[57] Ewels PA, Peltzer A, Fillinger S, Patel H, Alneberg J, Wilm A, Garcia MU, Di Tommaso P, Nahnsen S. 2020. The nf-core framework for community-curated bioinformatics pipelines. Nat Biotechnol 38:276–278. doi:10.1038/s41587-020-0439-x 32055031

[58] Di Tommaso P, Chatzou M, Floden EW, Barja PP, Palumbo E, Notredame C. 2017. Nextflow enables reproducible computational workflows. Nat Biotechnol 35:316–319. doi:10.1038/nbt.3820 28398311

[59] Callahan BJ, McMurdie PJ, Rosen MJ, Han AW, Johnson AJA, Holmes SP. 2016. DADA2: high-resolution sample inference from Illumina amplicon data. Nat Methods 13:581–583. doi:10.1038/nmeth.3869 27214047 PMC4927377

[60] Martin M. 2011. Cutadapt removes adapter sequences from high-throughput sequencing reads. EMBnet j 17:10. doi:10.14806/ej.17.1.200

[61] Quast C, Pruesse E, Yilmaz P, Gerken J, Schweer T, Yarza P, Peplies J, Glöckner FO. 2013. The SILVA ribosomal RNA gene database project: improved data processing and web-based tools. Nucleic Acids Res 41:D590–6. doi:10.1093/nar/gks1219 23193283 PMC3531112

[62] Parks DH, Chuvochina M, Rinke C, Mussig AJ, Chaumeil PA, Hugenholtz P. 2022. GTDB: an ongoing census of bacterial and archaeal diversity through a phylogenetically consistent, rank normalized and complete genome-based taxonomy. Nucleic Acids Res 50:D785–D794. doi:10.1093/nar/gkab776 34520557 PMC8728215

[63] Decelle J, Romac S, Stern RF, Bendif EM, Zingone A, Audic S, Guiry MD, Guillou L, Tessier D, Le Gall F, Gourvil P, Dos Santos AL, Probert I, Vaulot D, de Vargas C, Christen R. 2015. PhytoREF: a reference database of the plastidial 16S rRNA gene of photosynthetic eukaryotes with curated taxonomy. Mol Ecol Resour 15:1435–1445. doi:10.1111/1755-0998.12401 25740460

[64] Team Rs. 2019. RStudio: Integrated development for R. 1.2.5019. RStudio, Inc, Boston, MA.

[65] Lindh MV, Sjöstedt J, Andersson AF, Baltar F, Hugerth LW, Lundin D, Muthusamy S, Legrand C, Pinhassi J. 2015. Disentangling seasonal bacterioplankton population dynamics by high-frequency sampling. Environ Microbiol 17:2459–2476. doi:10.1111/1462-2920.12720 25403576

[66] Jan KMG, Serandour B, Walve J, Winder M. 2024. Plankton blooms over the annual cycle shape trophic interactions under climate change. Limnol Oceanogr Letters 9:209–218. doi:10.1002/lol2.10385

[67] Godrant A, Leynaert A, Moriceau B. 2024. A study of the influence of iron, phosphate, and silicate in Si uptake by two Synechococcus strains. Front Mar Sci 11. doi:10.3389/fmars.2024.1331333

[68] Phoenix VR, Adams DG, Konhauser KO. 2000. Cyanobacterial viability during hydrothermal biomineralisation. Chem Geol 169:329–338. doi:10.1016/S0009-2541(00)00212-6

[69] Lalonde SV, Konhauser KO, Reysenbach AL, Ferris FG. 2005. The experimental silicification of Aquificales and their role in hot spring sinter formation. Geobiology 3:41–52. doi:10.1111/j.1472-4669.2005.00042.x

[70] Hirota R, Hata Y, Ikeda T, Ishida T, Kuroda A. 2010. The silicon layer supports acid resistance of Bacillus cereus spores. J Bacteriol 192:111–116. doi:10.1128/JB.00954-09 19880606 PMC2798246

[71] Ikeda T. 2021. Bacterial biosilicification: a new insight into the global silicon cycle. Biosci Biotechnol Biochem 85:1324–1331. doi:10.1093/bbb/zbab069 33877302

[72] Ragueneau O, Tréguer P. 1994. Determination of biogenic silica in coastal waters: applicability and limits of the alkaline digestion method. Mar Chem 45:43–51. doi:10.1016/0304-4203(94)90090-6

[73] Krause JW, Brzezinski MA, Landry MR, Baines SB, Nelson DM, Selph KE, Taylor AG, Twiningf BS. 2010. The effects of biogenic silica detritus, zooplankton grazing, and diatom size structure on silicon cycling in the euphotic zone of the eastern equatorial Pacific. Limnology & Oceanography 55:2608–2622. doi:10.4319/lo.2010.55.6.2608

[74] Tang T, Kisslinger K, Lee C. 2014. Silicate deposition during decomposition of cyanobacteria may promote export of picophytoplankton to the deep ocean. Nat Commun 5:4143. doi:10.1038/ncomms5143 24920300

