# Supplementary material for: Seasonal dynamics and nutrient controls of biogenic silica in Baltic Sea surface microplankton and picoplankton communities
Source: Appl Environ Microbiol. 2025 Apr 28;91(5):e00676-25. doi: 10.1128/aem.00676-25 (PMC12094022; doi:10.1128/aem.00676-25)
Supplement: Supplemental material — Figures S1 to S5; Tables S1 to S3. [file aem.00676-25-s0001.docx]

**Supplementary Material**

**
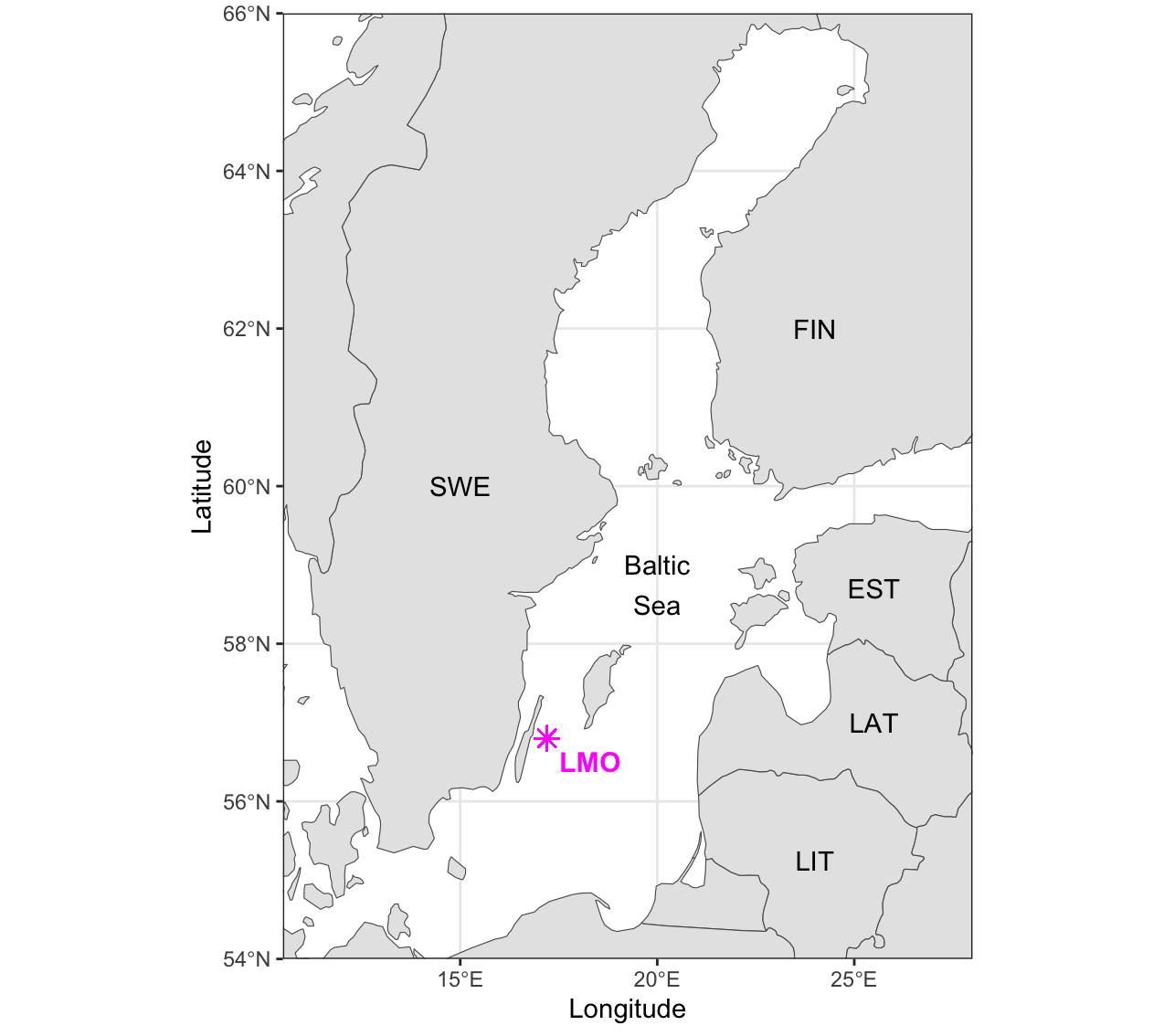
**

**Figure S1***.* Map of the Baltic Sea and surrounding area with the sampling location Linnaeus Microbial Observatory marked with an asterisk (*) and labeled with LMO.

**Figure S2.** Composition of major phytoplankton (>5 µm) taxonomic groups assessed using microscopy at the final timepoint (t65) of the four microcosm experiments (SPR, SUM, AUT, WIN) in all treatment bottles (*n=18*). Proportion of carbon biomass (%) is calculated from single microscopy samples.


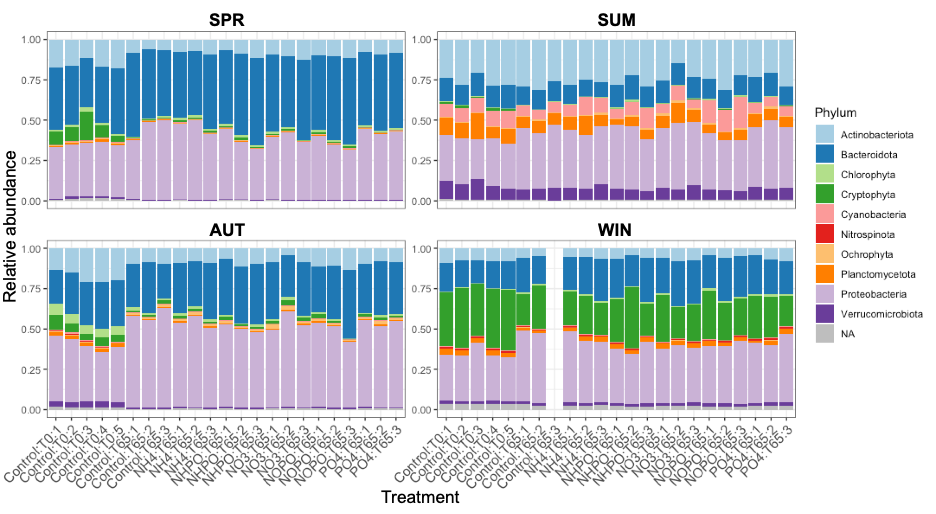


**Figure S3.** Relative abundance of picoplankton (0.22–3 µm) 16S rRNA gene amplicons at the initial (t0) and final timepoint (t65) of the four microcosm experiments (SPR, SUM, AUT, WIN). When confidence scores of ASV sequences assigned to chloroplasts in the SILVA 132 database were lower than confidence scores in the Phytoref database, then Phytoref taxonomic assignment was used for eukaryotic taxonomic assignment.


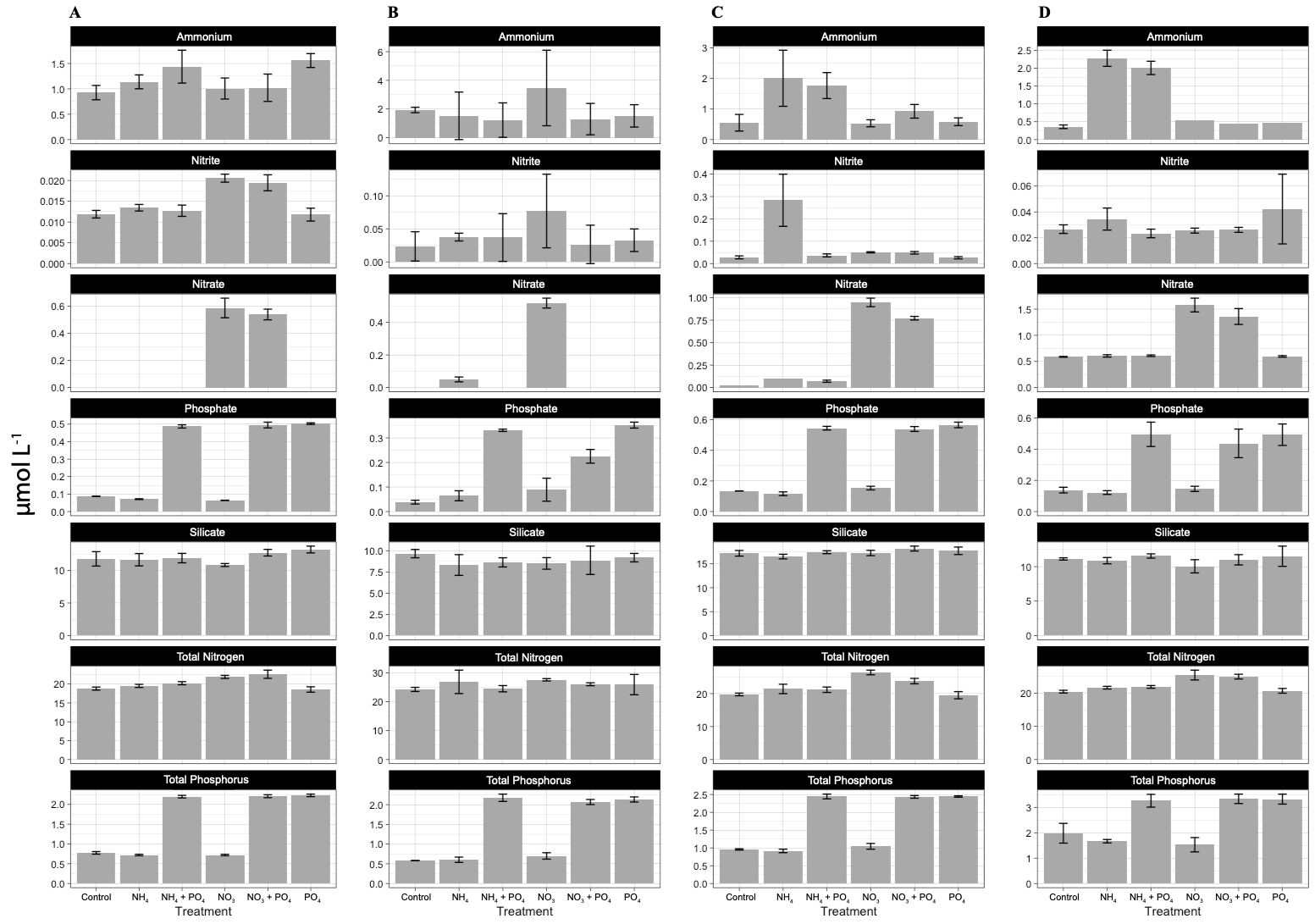


**Figure S4.** Nutrient concentrations measured at the final timepoint (t65) in microcosms (A) SPR, (B) SUM, (C) AUT, and (D) WIN. These nutrients include ammonium, nitrite, nitrate, phosphate, silicate, total nitrogen, and total phosphorus. Error bars indicate the ±SD of biological replicates (*n*=3). In the case of nitrate in A, B, and C, if all or the majority (2 out of 3) of the biological replicates had undetectable concentrations, then the average concentration was not shown on the bar plot.

**Figure S5.** Spearman correlations between microplankton bSi and the total carbon biomass of the various phytoplankton groups identified with microscopy at the final timepoint (t65) of the microcosm experiments (SPR, SUM, AUT, WIN). An asterisk (*) in the square indicates a statistically significant (p = <0.05) relationship.

**Table S1.** Biogenic (bSi) and lithogenic (LSi) silica measurements from LMO in picoplankton (0.22–3 µm) and microplankton (>3 µm) fractions separately, as well as total. bSi and LSi analysis was done on the same sample. The % LSi at each sampling date was calculated. ND indicates samples where the measured absorbance was zero.

| Date | Fraction | bSi (µmol L^-1^) | LSi (µmol L^-1^) | % LSi (LSi / bSi + LSi) |
| --- | --- | --- | --- | --- |
| June 15, 2021 | >3 µm | 0.154 | 0.017 |  |
|  | 0.22–3 µm | 0.010 | ND |  |
|  | Total | 0.164 | 0.017 | 9.4 |
| July 12, 2021 | >3 µm | 0.166 | 0.040 |  |
|  | 0.22–3 µm | 0.016 | ND |  |
|  | Total | 0.182 | 0.040 | 18.0 |
| September 7, 2021 | >3 µm | 0.637 | 0.040 |  |
|  | 0.22–3 µm | 0.063 | 0.009 |  |
|  | Total | 0.700 | 0.049 | 6.5 |
| September 21, 2021 | >3 µm | 0.665 | 0.057 |  |
|  | 0.22–3 µm | 0.074 | 0.019 |  |
|  | Total | 0.739 | 0.076 | 9.3 |
| October 5, 2021 | >3 µm | 0.767 | 0.037 |  |
|  | 0.22–3 µm | 0.095 | 0.017 |  |
|  | Total | 0.862 | 0.054 | 5.9 |

**Table S2.** Results of Kruskal-Wallis ANOVA test of average chlorophyll *a* values in microcosms SPR, SUM, AUT, WIN in picoplankton (0.22–3 µm) and microplankton (>3 µm) fractions. Chlorophyll *a (*µg L^-1^) averages were calculated in different timepoints (t0, t65) and treatments (Control, NH_4_, NH_4_ + PO_4_, NO_3_, NO_3_ + PO_4_, and PO_4_). Asterisks (*) indicate significant *p* values *(** ≤ 0.05, ** ≤ 0.01, *** ≤ 0.001).

| Microcosm | Fraction | Df | Chi-squared | *p* Value |
| --- | --- | --- | --- | --- |
| SPR | Microplankton | 5 | 13.815 | 0.017 (*) |
|  | Picoplankton | 5 | 14.146 | 0.015 (*) |
| SUM | Microplankton | 5 | 16.064 | 0.007 (**) |
|  | Picoplankton | 5 | 14.553 | 0.012 (*) |
| AUT | Microplankton | 5 | 9.718 | 0.084 |
|  | Picoplankton | 5 | 14.193 | 0.014 (*) |
| WIN | Microplankton | 5 | 7.085 | 0.214 |
|  | Picoplankton | 5 | 11.919 | 0.036 (*) |

**Table S3.**  Results of Kruskal-Wallis ANOVA test of average bSi values in microcosms SPR, SUM, AUT, WIN in picoplankton (0.22–3 µm) and microplankton (>3 µm) fractions. bSi *(*µmol L^-1^) averages were calculated in different timepoints (t0, t65) and treatments (Control, NH_4_, NH_4_ + PO_4_, NO_3_, NO_3_ + PO_4_, and PO_4_). Asterisks (*) indicate significant *p* values *(** ≤ 0.05, ** ≤ 0.01, *** ≤ 0.001).

| Microcosm | Fraction | Df | Chi-squared | *p* Value |
| --- | --- | --- | --- | --- |
| SPR | Microplankton | 5 | 13.749 | 0.017 (*) |
|  | Picoplankton | 5 | 10.825 | 0.055 |
| SUM | Microplankton | 5 | 13.468 | 0.019 (*) |
|  | Picoplankton | 5 | 12.375 | 0.03 (*) |
| AUT | Microplankton | 5 | 9.281 | 0.098 |
|  | Picoplankton | 5 | 5.913 | 0.315 |
| WIN | Microplankton | 5 | 7.503 | 0.186 |
|  | Picoplankton | 5 | 10.918 | 0.053 |
